# Low preoperative adiponectin levels are associated with postoperative systemic inflammatory response following major colorectal cancer surgery

**DOI:** 10.1186/s40635-026-00960-y

**Published:** 2026-08-26

**Authors:** Sonja Škiljić, Ines Šahinović, Gordana Kristek, Hrvoje Vinković, Ivana Haršanji-Drenjančević, Dino Budrovac, Bojan Trogrlić, Josip Kocur, Nenad Nešković, Slavica Kvolik

**Affiliations:** 1 Faculty of Medicine, University Josip Juraj Strossmayer, Osijek, Croatia; 2 Department of Anaesthesiology, Resuscitation and ICU, Osijek University Hospital, Osijek, Croatia; 3 Polyclinic Borzan, Osijek, Croatia; 4 Department of Abdominal Surgery, Osijek University Hospital, Osijek, Croatia; 5 Department of Orthopaedics and Traumatology, Osijek University Hospital, Osijek, Croatia; 6 International Medical Center Priora, Cepin, Croatia

**Keywords:** Adiponectin, Interleukin-6, Systemic Inflammatory Response Syndrome, Colorectal Surgery, Colorectal Cancer

## Abstract

**Background:**

Adiponectin is an adipokine secreted by adipose tissue that plays an important role in colorectal oncogenesis and the response to surgical stress through its anti-inflammatory effects. However, it remains unclear whether perioperative alterations in circulating adiponectin in patients undergoing colorectal cancer surgery are associated with early postoperative systemic inflammation. The aim of this study was to investigate the predictive value of perioperative adiponectin levels, interleukin-6 (IL-6), and standard inflammatory blood biomarkers for the development of postoperative systemic inflammatory response syndrome (SIRS) and complications following colorectal cancer surgery.

**Methods:**

This prospective observational study included 57 patients scheduled for open colorectal cancer surgery. Blood samples for determination of white blood cell count (WBC), differential leukocyte count, C-reactive protein (CRP), adiponectin, and IL-6 were collected preoperatively and at 24 and 72 h after surgery. The occurrence of surgical and non-surgical postoperative complications until hospital discharge was recorded. Patients were divided into two groups according to the presence of clinical and laboratory signs of SIRS within 72 h after surgery.

**Results:**

No statistically significant differences in demographic and clinical characteristics were observed between the SIRS and non-SIRS groups of patients. Patients who developed SIRS within 72 h after surgery had lower preoperative adiponectin levels, 10.1 (5.3–15.1) vs. 14.7 (9.6–20.9) mg/L, *p* = 0.02, and higher WBC, CRP, and CRP/albumin ratio. Multivariate logistic regression showed that among preoperative biomarkers, adiponectin was the only biomarker independently associated with postoperative SIRS, OR 0.91 (95% CI 0.83–0.99), *p* = 0.03. Using a cut-off serum level of preoperative adiponectin ≤ 7.5 mg/L, ROC analysis demonstrated sensitivity of 38.7% and specificity of 92.3% for predicting postoperative SIRS, with an AUC 0.673 (95% CI 0.534–0.812), *p* = 0.01. IL-6 measured 72 h after surgery demonstrated the highest predictive value for postoperative complications, with an AUC 0.736 (95% CI 0.593–0.880), *p* = 0.001, and sensitivity 66.7% and specificity 84.6% at a cut-off value ≥ 40.4 pg/mL.

**Conclusions:**

Low preoperative adiponectin levels are associated with the development of postoperative SIRS, but not postoperative complications, following colorectal cancer surgery.

## Introduction

Colorectal cancer (CRC) is one of the leading causes of cancer-related morbidity and mortality worldwide, and surgery remains the cornerstone of its management [1–4]. The host inflammatory response to both cancer and surgery represents one of the most intriguing areas of scientific and clinical research, as recent reports indicate its significant impact on disease progression and long-term prognosis [5]. Despite growing awareness of the importance of systemic inflammation in determining patient outcomes after CRC surgery, the reliability of existing inflammatory markers remains controversial. Currently, there is no single biomarker routinely used in clinical practice that demonstrates ideal performance with high sensitivity, specificity, and predictive value for monitoring early postoperative systemic inflammatory response syndrome (SIRS) and complications following major colorectal surgery [6]. Peripheral blood inflammatory indices, such as the neutrophil/lymphocyte ratio (NLR), platelet/lymphocyte ratio (PLR), and C-reactive protein (CRP)/albumin ratio, have been extensively investigated in recent years and have gained considerable popularity [7, 8]. Adiponectin is the most abundant adipokine secreted by adipose tissue [9]. Although epidemiological evidence suggests a strong association between dysregulation of the adiponectin system, inflammation, and CRC, its immunological role in surgical patients with CRC has not yet been fully elucidated. According to current evidence, it remains unclear whether perioperative alterations in circulating adiponectin concentrations are associated with early postoperative SIRS in patients undergoing surgery for CRC [10–13]. The aim of this study was to investigate the perioperative dynamics of adiponectin in relation to the development of postoperative SIRS and postoperative complications in patients undergoing CRC surgery and to compare its predictive value with established inflammatory biomarkers, including interleukin-6 (IL-6), CRP, NLR, PLR, and the CRP/albumin ratio.

## Methods

This prospective observational study was conducted from July 2023 to August 2025 at the Department of Abdominal Surgery and the Surgical Intensive Care Unit (ICU) of Osijek University Hospital, Croatia. The study was approved by the Ethics Committee of Osijek University Hospital (No. R1-4012/2023) and registered at ClinicalTrials.gov under the number NCT06057207. Written informed consent was obtained from all participants. The study included patients diagnosed with CRC who underwent elective open colorectal tumour resection with primary anastomosis and were subsequently admitted to the surgical ICU for 24 h in accordance with the institutional routine protocol for monitoring vital signs and detecting early adverse events. All patients received the same balanced general endotracheal anaesthesia. After induction with propofol (Propofol 1% MCT Fresenius, Fresenius Kabi, Bad Homburg, Germany), anaesthesia was maintained with mixture of sevoflurane (Sevoflurane, Baxter Healthcare Ltd., Thetford, Norfolk, England), oxygen and air, rocuronium bromide (Esmeron, NV Organon, Amsterdam, Netherlands), and fentanyl (Fentanyl Janssen, Janssen-Cilag, Beerse, Belgium). Postoperatively, all patients received the same multimodal analgesic regimen consisting of tramadol (Tramal, Stada Arzneimittel AG, Bad Vilbel, Germany) and paracetamol (Paracetamol, B. Braun, Melsungen, Germany), with additional meperidine boluses (Dolsin, Pharma a.s., Prague, Czech Republic). Clinical and laboratory indicators of systemic inflammation were monitored postoperatively. In addition to standard inflammatory biomarkers (white blood cell count (WBC), differential leukocyte count, and CRP), IL-6 and adiponectin concentrations were measured preoperatively and at 24 and 72 h after surgery. Patients were divided into two groups, SIRS and non-SIRS, according to the presence of clinical and laboratory signs of postoperative SIRS. The development of SIRS within the first 72 h after surgery was defined as the presence of at least two of the following four major criteria: tachycardia > 90 beats/min, body temperature > 38 °C or < 36 °C, WBC > 12,000/mm³ or < 4,000/mm³, and hyperventilation (> 20 breaths/min) or a decrease in the partial pressure of arterial CO_2_ < 4.3 kPa. In addition, postoperative CRP levels > 50 mg/L and procalcitonin (PCT) levels > 0.5 µg/L, in the presence of at least one clinical criterion, were considered indicative of SIRS. In cases of borderline CRP and PCT values and the presence of fewer than two clinical criteria, an arterial lactate level > 2 mmol/L persisting for more than four hours within the first 72 postoperative hours was used to confirm SIRS [14–16]. Postoperative follow-up was conducted throughout the entire hospitalization period until discharge or death. The length of hospital stay, and the occurrence of postoperative complications were recorded. Complications were categorized as surgical (local infection, drain leak, anastomotic dehiscence, wound disruption, ileus, or bleeding) and non-surgical (delirium, sepsis, pneumonia, respiratory failure, renal failure, or cardiovascular failure). Participants without histopathological confirmation of colorectal cancer were subsequently excluded from the study. Additionally, patients were excluded if they were younger than 18 years old, had an emergency surgical condition, suspected infection, underwent laparoscopic colorectal surgery, had a body mass index < 18.5 kg/m² or > 40 kg/m², had autoimmune disease, inflammatory bowel disease, liver cirrhosis, were receiving corticosteroid or immunomodulatory therapy, experienced massive intraoperative bleeding [17], or refused to participate in the study. Based on previous studies [18–20], assuming an effect size ranging from d = 0.9 to d = 1.05 for differences in adiponectin levels between two independent groups, with a significance level of 0.05 and statistical power of 0.80, the minimum required sample size was calculated to be 42 participants. After accounting for an additional 10% of participants to compensate for potential attrition, the minimum required sample size for the study was 47 participants (calculated using G*Power version 3.1.9.4) [21].

### Determination of adiponectin and IL-6 concentrations

Blood samples were collected preoperatively and at 24 and 72 h after surgery. For the determination of conventional laboratory parameters, samples were obtained from peripheral venous and arterial blood. For the measurement of serum adiponectin and IL-6 concentrations, an additional 5 mL of peripheral venous blood was collected into tubes containing thrombin as a clot activator (BD Vacutainer, Becton, Dickinson and Company, Franklin Lakes, New York, USA).

Serum adiponectin concentrations were determined using a commercially available sandwich enzyme-linked immunosorbent assay (ELISA) kit according to the manufacturer’s instructions (Adiponectin ELISA Kit, Thermo Fisher Scientific Inc., Waltham, Massachusetts, USA). Serum samples were diluted 1:3 with phosphate buffer (pH 7.2). The assay is based on the formation of an antibody–antigen–horseradish peroxidase-conjugated antibody complex, with colour development following the addition of tetramethylbenzidine (TMB) substrate. Absorbance was measured at 450 nm, and concentrations were calculated from a calibration curve. Analyses were performed using an EtiMax ELISA processor (DiaSorin, Saluggia, Italy).

Serum IL-6 concentrations were measured using an electrochemiluminescence immunoassay (ECLIA) on a Cobas e402 immunochemistry analyser (Roche Diagnostics GmbH, Mannheim, Germany), according to the manufacturer’s instructions (ELECSYS IL-6 Immunoassay, Roche, Basel, Switzerland). The assay is based on the formation of a biotinylated antibody–IL-6–ruthenium-labelled antibody immunocomplex, followed by magnetic separation and electrochemiluminescent signal detection. Signal intensity is directly proportional to the IL-6 concentration and was quantified using a calibration curve.

### Statistical analysis

Categorical data were presented as absolute and relative frequencies, and continuous variables as medians with interquartile ranges (IQR). Continuous variables were tested for normality using the Shapiro-Wilk test. Differences between categorical variables were assessed using Fisher’s exact test, and differences between continuous variables were assessed using the Mann–Whitney U test. Univariable and multivariable logistic regression analysis (backward Wald method) was used to evaluate the effect of independent predictors on the occurrence of SIRS and postoperative complications. Variables showing a potential association in the univariable model or considered clinically relevant and non-collinear were included in the multivariable model. Receiver operating characteristic (ROC) curve analysis with 95% confidence intervals (CI) was used to assess the predictive performance of individual biomarkers for predicting SIRS and postoperative complications. Optimal cut-off values were determined using the Youden index. Statistical significance was defined as α = 0.05, and all p values were two-sided. Data were analysed using IBM SPSS Statistics, version 29 (IBM Corp., Armonk, NY, USA) and JASP, version 0.19.5 (JASP Team, 2024).

## Results

A total of 57 patients with colorectal cancer who underwent open surgical resection were included in the study, including 33 men (57.9%) and 24 women (42.1%). The tumour was located in the colon in 37 patients (64.9%) and in the rectosigmoid colon in 20 patients (35.1%). Within the first 72 h postoperatively, 31 patients (54.4%) fulfilled the clinical criteria of SIRS. The demographic and clinical characteristics of the study cohort are summarized in Table 1.

**Table 1:** Demographic and clinical characteristics of the study cohort

	Median (IQR) or n (%)				*p* ^*^
	All patients (*N* = 57)		SIRS (*N* = 31)	Non-SIRS (*N* = 26)	
Age (years)	70 (66–74)		68 (64–73)	72 (68–76)	0.04
Sex (male)	33 (57.9)		19 (61.3)	14 (53.8)	0.60
BMI (kg/m^2^)	26.4 (23.9–29.8)		26.7 (24.3–31.7)	24.7 (23.4–28.2)	0.06
Cancer region Colon Rectosigmoid	37 (64.9) 20 (35.1)		19 (61.3) 12 (38.7)	18 (69.2) 8 (30.8)	0.59
ASA II III	17 (29.8) 40 (70.2)		11 (35.5) 20 (64.5)	6 (23.1) 20 (76.9)	0.39
Comorbidities Hypertension Diabetes mellitus Cardiovascular disease Metabolic syndrome	41 (71.9) 17 (29.8) 9 (15.8) 13 (22.8)		22 (71.0) 10 (32.3) 4 (12.9) 9 (29.0)	19 (73.1) 7 (26.9) 5 (19.2) 4 (15.4)	> 0.99 0.74 0.72 0.34
Preoperative chemotherapy	14 (24.6)		7 (22.6)	7 (26.9)	0.76
Length of hospital stay (days)	7 (6–10)		8 (6–12)	7 (6–8)	0.04

* Mann–Whitney U test for continuous variables and Fisher’s exact test for categorical variables between SIRS and Non-SIRS groups; SIRS: systemic inflammatory response syndrome developing within the first 72 h postoperatively; BMI: body mass index; ASA: American Society of Anesthesiologists classification

### Inflammatory biomarkers according to postoperative SIRS

**Fig. 1:**
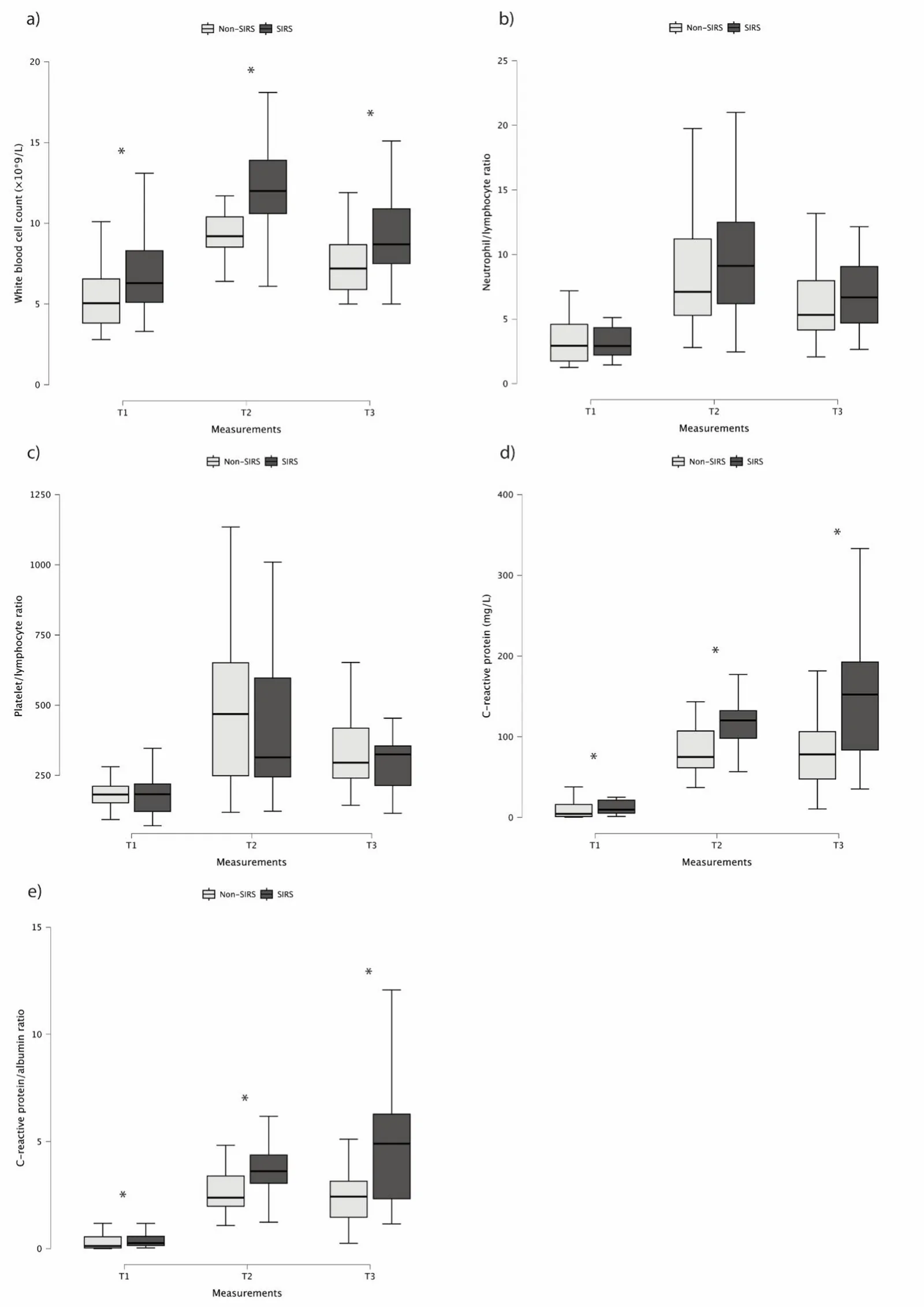
Differences in white blood cell count (**a**), neutrophil/lymphocyte ratio (**b**), platelet/lymphocyte ratio (**c**), C-reactive protein (**d**), and C-reactive protein/albumin ratio (**e**) according to the development of postoperative SIRS. SIRS: systemic inflammatory response syndrome; T1: preoperative measurement; T2: 24 h postoperative; T3: 72 h postoperative; * *p* < 0.05 (Mann-Whitney U test)

Patients who developed SIRS had significantly higher preoperative WBC counts, 6.3 (5.1–8.3) vs. 5.05 (3.7–6.6) ×10^9^/L, *p* = 0.03, CRP levels, 9.7 (5.4–25.1) vs. 4.5 (1.2–17.2) mg/L, *p* = 0.03, and CRP/albumin ratio, 0.26 (0.13–0.65) vs. 0.12 (0.03–0.56), *p* = 0.03, than patients without SIRS. At 24 and 72 h postoperatively, standard inflammatory biomarkers remained significantly higher in the SIRS group, while NLR and PLR showed no significant differences between the groups at any time point (Fig. 1).

**Fig. 2:**
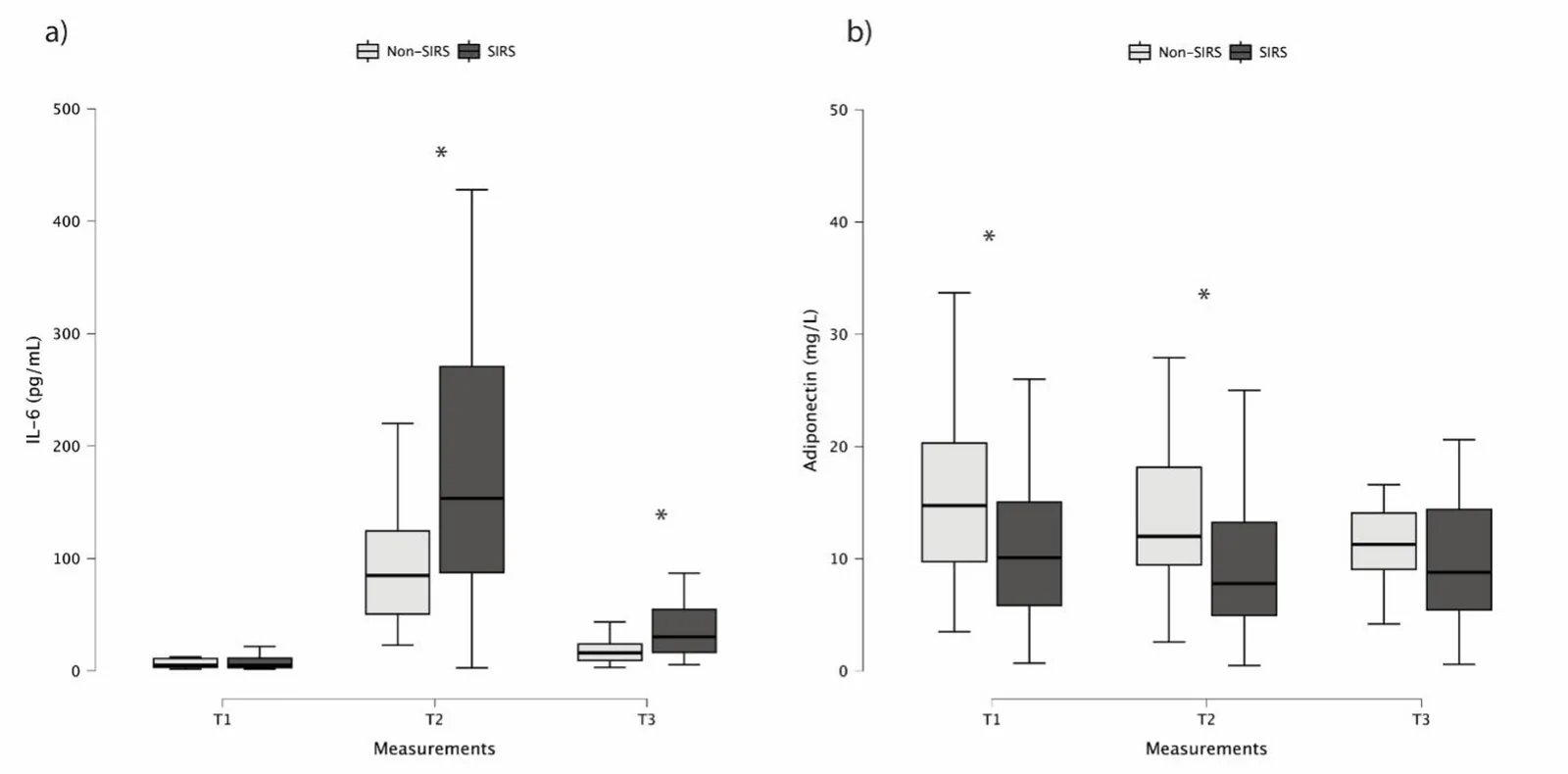
Differences in IL-6 (**a**) and adiponectin levels (**b**) according to the development of postoperative SIRS. SIRS: systemic inflammatory response syndrome; T1: preoperative measurement; T2: 24 h postoperative; T3: 72 h postoperative; * *p* < 0.05 (Mann-Whitney U test)

Preoperative serum IL-6 levels did not differ significantly between the groups, while preoperative serum adiponectin levels were lower in patients who developed postoperative SIRS, 10.1 (5.3–15.1) vs. 14.7 (9.6–20.9) mg/L, *p* = 0.02. At 24 h postoperatively, patients with SIRS had significantly higher IL-6 and lower adiponectin levels. By 72 h, IL-6 levels remained significantly elevated in the SIRS group, 30.3 (15.8–55.1) vs. 16.3 (9.1–28.3) pg/mL, *p* = 0.02. No significant difference in late adiponectin levels was observed between the groups, 8.8 (5.3–15.6) vs. 11.3 (9.0–14.7) mg/L, *p* = 0.08 (Fig. 2).

### Predictive value of IL-6 and adiponectin for the development of postoperative SIRS

**Fig. 3:**
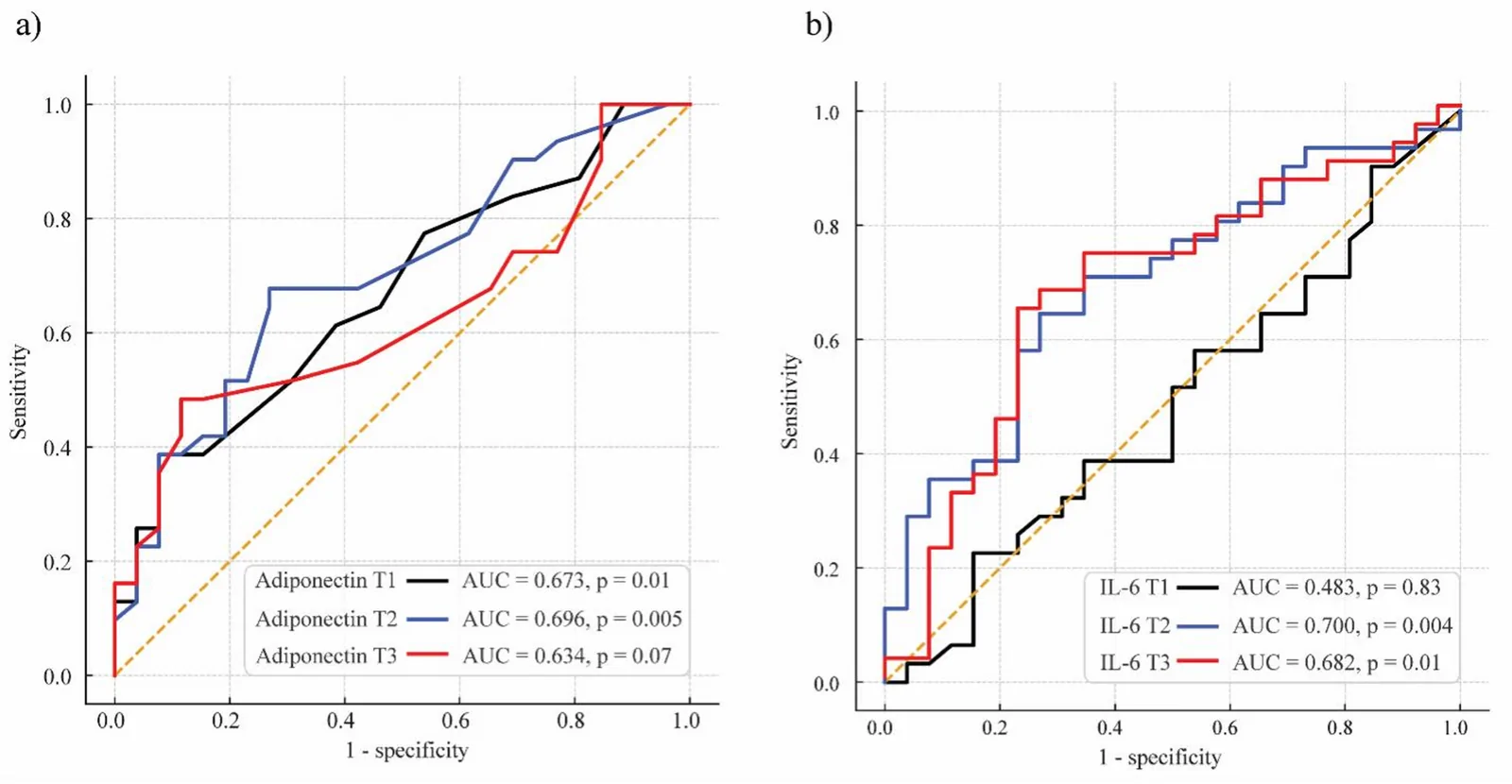
ROC curves of adiponectin (**a**) and IL-6 (**b**) for predicting postoperative SIRS. T1: preoperative measurement; T2: 24 h postoperative; T3: 72 h postoperative; AUC: area under the curve

ROC curve analysis was performed to assess the ability of adiponectin and IL-6 levels to distinguish patients with and without postoperative SIRS. Preoperative adiponectin yielded an AUC of 0.673 (95% CI 0.534–0.812), *p* = 0.01. Using an optimal cut-off value of ≤ 7.5 mg/L, the sensitivity and specificity for predicting postoperative SIRS were 38.7% and 92.3%, respectively. At 24 h postoperatively, adiponectin showed an AUC of 0.696 (95% CI 0.559–0.833), *p* = 0.005, with an optimal cut-off value of ≤ 10.46 mg/L, sensitivity of 67.7%, and specificity of 73.1%. At 72 h postoperatively, the AUC was 0.634 (95% CI 0.488–0.780), *p* = 0.07 (Fig. 3a). Preoperative IL-6 was not significantly associated with postoperative SIRS (AUC 0.483; 95% CI 0.330–0.635), *p* = 0.83. Significant ROC results were observed at 24 and 72 h postoperatively, with cut-off values of ≥ 115.2 pg/mL and ≥ 25.4 pg/mL, respectively. The corresponding sensitivities were 64.5% at both time points, while specificities were 73.1% and 76.9%, respectively (Fig. 3b).

In the univariable logistic regression analysis, adiponectin, IL-6, WBC, CRP, and the CRP/albumin ratio measured preoperatively and postoperatively were evaluated as biomarkers associated with postoperative SIRS. Preoperatively, significant associations were observed for adiponectin and WBC. At 24 h postoperatively, significant results were observed for all evaluated biomarkers, while at 72 h significance was observed for WBC, CRP, and the CRP/albumin ratio (Table 2).

**Table 2:** Univariable logistic regression analysis of factors associated with postoperative SIRS

Independent variables	β	Wald	*p*	Odds ratio (95% CI)
Preoperative				
Adiponectin IL-6 WBC CRP CRP/albumin	-0.085 -0.013 0.288 0.017 0.428	5.034 0.159 4.759 2.120 1.473	0.02 0.69 0.03 0.14 0.22	0.92 (0.85–0.99) 0.99 (0.92–1.05) 1.33 (1.03–1.72) 1.02 (0.99–1.04) 1.53 (0.77–3.06)
24 h postoperative				
Adiponectin IL-6 WBC CRP CRP/albumin	-0.106 0.006 0.669 0.029 0.642	5.696 4.473 11.227 9.031 6.549	0.02 0.03 < 0.001 0.003 0.01	0.90 (0.83–0.98) 1.01 (1.00–1.01) 1.95 (1.33–2.87) 1.03 (1.03–1.05) 1.90 (1.16–3.11)
72 h postoperative				
Adiponectin IL-6 WBC CRP CRP/albumin	-0.091 0.013 0.359 0.011 0.301	3.565 1.935 6.634 6.244 5.572	0.06 0.16 0.01 0.01 0.02	0.91 (0.83–1.00) 1.01 (0.99–1.04) 1.43 (1.09–1.88) 1.01 (1.00–1.02) 1.35 (1.05–1.73)

β: regression coefficient; CI: confidence interval; WBC: white blood cells; CRP: C-reactive protein

In the multivariable logistic regression model adjusted for comorbidities (hypertension, diabetes, cardiovascular disease, and metabolic syndrome), preoperative adiponectin was the only variable significantly associated with postoperative SIRS, OR 0.91 (95% CI 0.83–0.99), *p* = 0.03. In the model including biomarkers measured 24 h after surgery, only WBC was significantly associated with postoperative SIRS, OR 2.63 (95% CI 1.52–4.53), *p* < 0.001. Both models were statistically significant overall and showed classification accuracies of 59.6% and 84.2%, respectively (Table 3).

**Table 3:** Multivariable logistic regression analysis of factors associated with postoperative SIRS

Independent variables	β	Wald	p*	Odds ratio (95% CI)
Preoperative				
Adiponectin WBC Constant	-0.097 0.288 -0.293	4.417 3.784 0.069	0.03 0.05 0.79	0.91 (0.83–0.99) 1.33 (0.99–1.78)
24 h postoperative				
Adiponectin WBC Constant	-0.134 0.966 -9.236	3.722 12.054 9.606	0.05 < 0.001 0.002	0.87 (0.76–1.00) 2.63 (1.52–4.53)
72 h postoperative				
Adiponectin WBC CRP/albumin Constant	-0.123 0.293 0.338 -1.615	3.268 3.039 3.730 1.397	0.07 0.08 0.05 0.24	0.88 (0.77–1.01) 1.34 (0.96–1.86) 1.40 (0.99–1.98)

*Adjusted for hypertension, diabetes, cardiovascular disease, and metabolic syndrome; β: regression coefficient; CI: confidence interval; WBC: white blood cells; CRP: C-reactive protein

### Inflammatory biomarkers according to postoperative complications

A total of 18 patients (31.6%) developed at least one surgical and/or non-surgical postoperative complication, of whom eight (14%) experienced both types of complications (Table 4).

**Table 4:** Distribution of surgical and non-surgical postoperative complications

Postoperative complications*	*N* (%)
Surgical Local infection Drain leak Anastomosis disruption Wound disruption Ileus Bleeding	10 (17.5) 4 (7.0) 2 (3.5) 2 (3.5) 5 (8.8) 1 (1.8)
Non-surgical Sepsis Delirium Pneumonia Respiratory failure Renal failure	1 (1.8) 4 (7.0) 7 (12.3) 1 (1.8) 7 (12.3)

* Some patients experienced more than one surgical or non-surgical postoperative complication

**Fig. 4:**
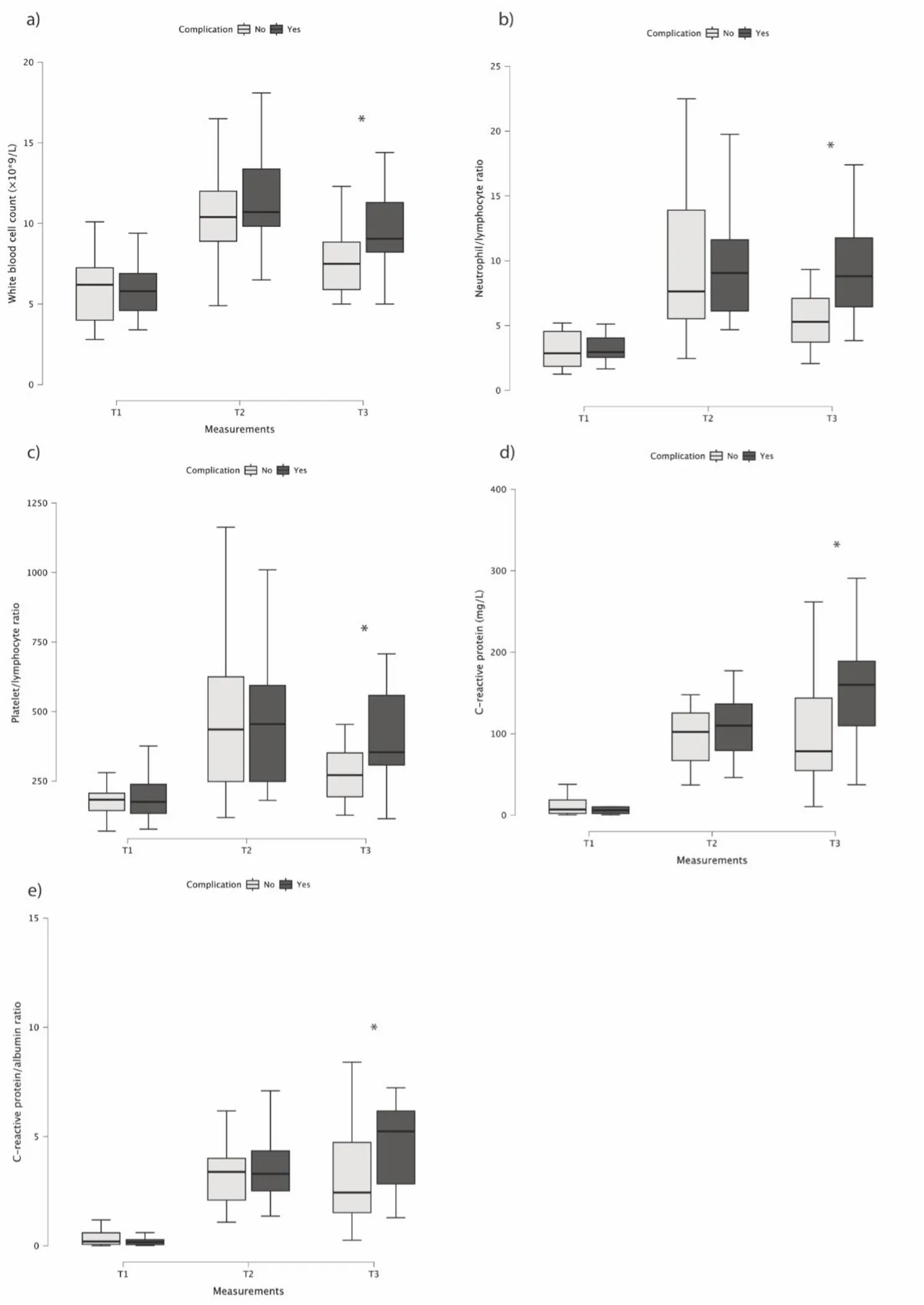
Differences in white blood cell count (**a**), neutrophil/lymphocyte ratio (**b**), platelet/lymphocyte ratio (**c**), C-reactive protein (**d**), and C-reactive protein/albumin ratio (**e**) according to the development of postoperative complications. SIRS: systemic inflammatory response syndrome; T1: preoperative measurement; T2: 24 h postoperative; T3: 72 h postoperative; * *p* < 0.05 (Mann-Whitney U test)

Compared with patients without complications, those who developed postoperative complications had significantly higher levels of inflammatory biomarkers, including NLR, PLR, and the CRP/albumin ratio at 72 h after surgery (Fig. 4).

**Fig. 5:**
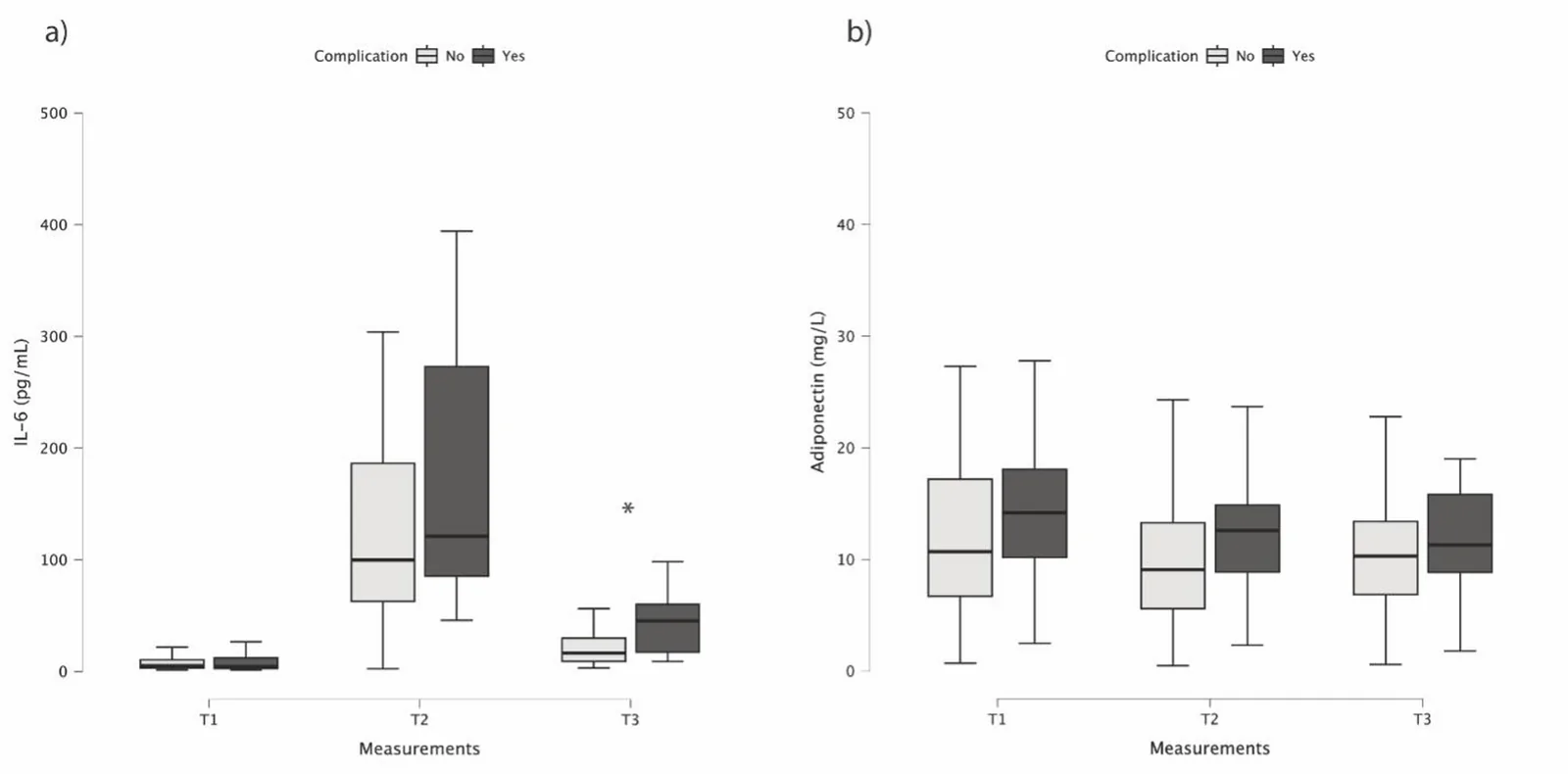
Differences in IL-6 (**a**) and adiponectin concentration (**b**) according to the development of postoperative complications. T1: preoperative measurement; T2: 24 h postoperative; T3: 72 h postoperative; * *p* < 0.05 (Mann-Whitney U test)

IL-6 levels were also significantly higher at 72 h after surgery in patients who developed postoperative complications, 45.4 (16.3–64.6) vs. 16.7 (9.2–30.3) pg/mL, *p* = 0.004. In contrast, no significant differences in serum adiponectin concentrations were observed between the two groups at any of the measured time points (Fig. 5).

ROC curve analysis showed that IL-6 measured 72 h after surgery had the highest predictive value for postoperative complications, with an AUC of 0.736 (95% CI 0.593–0.880), *p* = 0.001. At a cut-off value of ≥ 40.4 pg/mL, IL-6 demonstrated 66.7% sensitivity and 84.6% specificity in predicting postoperative complications.

Univariable logistic regression showed that higher levels of IL-6, WBC, CRP, and the CRP/albumin ratio measured 72 h after surgery were significantly associated with postoperative complications (Table 5).

**Table 5:** Univariable logistic regression analysis of factors associated with postoperative complications

Independent variables	β	Wald	*p*	Odds ratio (95% CI)
Preoperative				
Adiponectin IL-6 WBC CRP CRP/albumin	0.018 -0.008 0.031 -0.007 -0.251	0.254 0.047 0.071 0.362 0.462	0.62 0.83 0.79 0.55 0.49	1.02 (0.95–1.09) 0.99 (0.92–1.06) 1.03 (0.82–1.29) 0.99 (0.97–1.02) 0.78 (0.34–1.60)
24 h postoperative				
Adiponectin IL-6 WBC CRP CRP/albumin	0.017 0.003 0.095 0.004 0.077	0.228 3.392 1.021 0.291 0.166	0.63 0.07 0.31 0.59 0.68	1.02 (0.95–1.09) 1.00 (1.00–1.01) 1.09 (0.91–1.32) 1.00 (0.99–1.02) 1.08 (0.74–1.56)
72 h postoperative				
Adiponectin IL-6 WBC CRP CRP/albumin	0.010 0.022 0.263 0.010 0.241	0.049 4.609 5.035 5.932 4.914	0.82 0.03 0.03 0.02 0.03	1.01 (0.92–1.10) 1.02 (1.00–1.04) 1.30 (1.03–1.64) 1.01 (1.00–1.02) 1.27 (1.03–1.57)

β: regression coefficient; CI: confidence interval; WBC: white blood cells; CRP: C-reactive protein

In the multivariable logistic regression model adjusted for comorbidities, none of the preoperative or early postoperative biomarkers were significantly associated with postoperative complications. Only 72 h after surgery did IL-6 and WBC show a tendency toward association with postoperative complications. However, this did not reach statistical significance in our cohort (Table 6).

**Table 6:** Multivariable logistic regression analysis of factors associated with postoperative complications

Independent variables	β	Wald	p*	Odds ratio (95% CI)
Preoperative				
No significant predictor				
24 h postoperative				
No significant predictor				
72 h postoperative				
IL-6 WBC Constant	0.022 0.247 -5.837	2.947 3.008 9.908	0.08 0.08 0.002	1.02 (0.99–1.05) 1.28 (0.97–1.69)

*Adjusted for hypertension, diabetes, cardiovascular disease, and metabolic syndrome; β: regression coefficient; CI: confidence interval; WBC: white blood cells; CRP: C-reactive protein

## Discussion

This study demonstrated that, among analysed preoperative biomarkers, only low preoperative adiponectin levels were significant predictors of postoperative SIRS following CRC surgery. The levels of other inflammatory biomarkers became significant only after surgery. Even after adjustment for relevant covariates, preoperative serum adiponectin was the only biomarker that remained independently associated with the development of postoperative SIRS in our cohort. Various inflammatory biomarkers so far are used to evaluate the surgical stress response and postoperative, inflammation-related complications following major colorectal surgery. Among these, WBC and CRP have been widely used and established biomarkers of active systemic inflammation in clinical practice [22]. In our study, multivariate analysis showed that the preoperative WBC count was not a significant standalone indicator of postoperative SIRS. This result is likely attributable to the confounding influence of other covariates, supporting prior assertions that the WBC count functions as a nonspecific inflammatory marker, susceptible to individual variations and underlying malignancy, rather than a reliable independent predictor of acute systemic inflammation following surgery [23–27].

It is known that during colorectal resections, traumatised cells stimulate the immune system to release pro-inflammatory cytokines such as TNF-α, IL-1, IL-6, and IL-8 through damage-associated molecular patterns (DAMPs) [28]. Simultaneously, compensatory anti-inflammatory pathways keep this process locally confined [29]. Under physiological conditions, a certain extent of postoperative inflammation is predictable and proportional to surgical stress, ideally leading to the natural healing process [30]. Exaggerated activation of a pro-inflammatory state following surgery is accompanied by complex neuroendocrine and immunometabolic changes driven by the release of inflammatory mediators affecting the whole body [31, 32]. While other biomarkers primarily reflect the postoperative inflammatory response after the release of pro-inflammatory mediators, adiponectin, beyond its principal metabolic role, also exerts intrinsic anti-inflammatory effects through the inhibition of various pro-inflammatory cytokines [33, 34]. Accordingly, higher preoperative adiponectin concentrations may modulate the postoperative inflammatory response by attenuating its proinflammatory component. In patients with lower preoperative adiponectin levels, this inhibitory effect may be diminished or absent. Previous studies examining the role of adiponectin in the postoperative inflammatory response in CRC have shown inconsistent results. Asgeirsson et al. highlighted the potential role of adiponectin as a marker of operative stress in a cohort of 44 patients undergoing colectomy [35]. In contrast, Ekström et al. reported no association between adiponectin and the early phase of acute systemic inflammation [36]. A more comprehensive evaluation of the relationship between adiponectin and the surgical stress-induced inflammatory response during major colorectal surgery was provided by a prospective randomized clinical trial conducted by Shi et al. Their study demonstrated an inverse association between perioperative circulating adiponectin levels and inflammatory biomarkers, including IL-6 and TNF-α, as well as markers of oxidative stress, in the context of different surgical approaches. However, the relatively small sample size limited the ability to perform multivariable regression analyses, thereby precluding a more robust assessment of the independent relationship between adiponectin and the development of systemic inflammatory response [37].

Previous studies examining the association between adiponectin and postoperative complications have reported inconsistent findings, with some demonstrating a significant association and others finding no relationship [38–40]. Interpretation and comparison of studies investigating adiponectin and postoperative outcomes following colorectal surgery are complicated by the lack of a standardized approach to defining postoperative morbidity. Although the Clavien-Dindo classification is widely accepted, recent evidence suggests that it may underestimate the clinical impact of lower-grade complications that nevertheless prolong hospital stay and increase healthcare costs [41, 42]. Furthermore, postoperative systemic inflammation has emerged as an independent determinant of recovery and long-term oncological outcomes, irrespective of complication severity [43, 44]. Chelazzi et al. reported an association between preoperative adiponectin levels and major postoperative complications in a pilot study of 12 patients undergoing colorectal cancer surgery [45]. In contrast, despite a substantially larger cohort, we did not observe an association between perioperative circulating adiponectin levels and postoperative complications. This discrepancy may partly reflect methodological differences between studies, including differences in the definition and classification of postoperative morbidity. However, patients who developed postoperative SIRS experienced a longer hospital stay regardless of the occurrence of postoperative complications. Given accumulating evidence that the magnitude of postoperative systemic inflammation may influence long-term oncological outcomes in colorectal cancer patients [46–48], our findings suggest that postoperative SIRS may represent a clinically relevant adverse postoperative event in its own, with preoperative circulating adiponectin levels potentially serving as a predictive marker. Early identification of patients at increased risk of postoperative SIRS may facilitate risk stratification, support tailored perioperative and postoperative care, and potentially improve clinical outcomes in patients undergoing colorectal cancer surgery.

In contrast to adiponectin, IL-6 was not independently associated with postoperative SIRS. However, IL-6 and WBC measured 72 h after surgery were the only biomarkers that showed a tendency toward a significant association with postoperative complications. A recent study by Procházka et al., conducted in 115 patients undergoing colorectal surgery, demonstrated the superiority of early postoperative IL-6 over CRP in predicting severe postoperative complications, including infectious ones [49]. Our results are consistent with the study by Rettig et al. which showed that early postoperative, but not preoperative, IL-6 levels were associated with complications following colorectal surgery. In that study, the occurrence of SIRS was not independently associated with postoperative complications after multivariable analysis, suggesting that IL-6 may represent a more specific marker of inflammation [50]. Indeed, IL-6 has most often been identified in the literature as a biomarker of postoperative infections following colorectal surgery rather than a marker of postoperative systemic inflammation itself. Boersema et al. also confirmed the superiority of early postoperative IL-6 levels over CRP in detecting infectious complications after colorectal surgery, but not non-infectious ones [51]. According to the literature, infectious complications after colorectal surgery most commonly develop between the eighth and tenth postoperative days [52]. Most inflammatory biomarkers demonstrate a statistically significant association with infectious complications only around 72 h after surgery [53]. Consistent with these observations, our study showed that among all analysed biomarkers, serum IL-6 levels ≥ 40.4 pg/mL measured 72 h after surgery showed the highest predictive value for postoperative complications. Current evidence suggest that adiponectin appears to act as a modulator of the immune response to surgical stress, rather than a simple downstream marker, as shown by higher adiponectin levels and concurrently lower circulating IL‑6, TNF‑α, IL‑1β and oxidative stress markers in patients undergoing laparoscopic compared with open colorectal surgery [37]. In contrast, IL‑6 is a prototypical effector cytokine of the systemic inflammatory response, rising in proportion to the magnitude of surgical stress and correlating with postoperative complications and recovery, and is therefore more appropriately interpreted as a consequence and biomarker of an established inflammatory response rather than its primary modulator [33, 34, 54–56].

Peripheral blood inflammatory indices such as NLR, PLR, and the CRP/albumin ratio have been extensively investigated in recent years in the context of the inflammatory response associated with CRC, which plays an important role in tumour progression and long-term survival [57, 58]. Likewise, several studies have demonstrated their value in predicting complications following colorectal surgery, particularly anastomotic leakage. A retrospective analysis by Zhang JX and Xu N. et al. demonstrated the predictive role of NLR and PLR for long-term prognosis in surgical CRC patients who developed postoperative anastomotic leakage [59]. Similarly, Paliogiannis et al., in a retrospective analysis of a large cohort, confirmed the predictive value of postoperative NLR and PLR for anastomotic complications [60]. Donlon et al., in a retrospective study, showed the usefulness of the early postoperative CRP/albumin ratio in predicting infectious complications following colorectal surgery [61]. In contrast, our results showed that perioperatively determined NLR, PLR, and the CRP/albumin ratio were not associated with the onset of postoperative systemic inflammation nor with postoperative complications.

To our knowledge, this is the first study to demonstrate the predictive role of perioperative circulating adiponectin levels, alongside novel inflammatory biomarkers, in the context of early postoperative systemic inflammation and short-term complications following open colorectal cancer surgery. Another strength of this study is the homogeneity of the cohort, as all patients underwent surgery using an open (laparotomy) approach, thereby avoiding potential differences in inflammatory response associated with laparoscopic techniques [62]. In addition, all included patients had histopathologically confirmed colorectal cancer. Finally, the prospective design of the study represents an additional methodological strength compared with most previously published studies in this field.

Our study also has several limitations. First, this was a single-centre observational study with a relatively small sample size. The relatively small sample size and limited number of SIRS events should be considered when interpreting the multivariable analysis. Consequently, the identified independent associations should be regarded as exploratory and hypothesis-generating, requiring validation in larger prospective cohorts. Also, patients were classified according to the development of postoperative SIRS, which may reflect differences in underlying inflammatory phenotypes and could have influenced postoperative biomarker profiles. However, SIRS classification was based on established clinical criteria rather than laboratory biomarkers, and the observed differences in preoperative biomarkers levels preceded the development of postoperative SIRS. Finally, we used a composite endpoint of postoperative complications that included both infectious and non-infectious events and inflammatory biomarkers were measured only preoperatively and at 24 and 72 h postoperatively. Extending the observation period to later postoperative days throughout the entire hospitalization would likely provide a more detailed understanding of the dynamics of inflammatory parameters in relation to systemic inflammation and postoperative complications.

## Conclusion

Preoperative serum adiponectin levels were independently associated with the development of postoperative SIRS following major colorectal cancer surgery, indicating a potential role for adiponectin as an early biomarker for identifying patients at increased risk of postoperative systemic inflammatory response. In contrast, perioperative serum levels of IL-6, CRP, NLR, PLR, and the CRP/albumin ratio were not independently associated with postoperative systemic inflammation in our cohort. Further studies involving larger cohorts and longer follow-up periods are needed to confirm the role of preoperative circulating adiponectin as a potential biomarker of postoperative systemic inflammation and long-term outcomes in patients undergoing surgery for colorectal cancer.

## Data Availability

The data presented in this study are available on request from the corresponding author.

## References

[1] Roshandel G, Ghasemi-Kebria F, Malekzadeh R, Colorectal Cancer (2024) Epidemiology, Risk Factors, and Prevention. Cancers (Basel) 16(8):1530. 10.3390/cancers16081530 38672612 10.3390/cancers16081530 PMC11049480

[2] The Lancet Gastroenterology H (2025) The rise in early-onset colorectal cancer: now a global issue. Lancet Gastroenterol Hepatol 10(2):95. 10.1016/S2468-1253(24)00441-2 39805285 10.1016/S2468-1253(24)00441-2

[3] Gustafsson UO, Rockall TA, Wexner S, How KY, Emile S, Marchuk A et al (2025) Guidelines for perioperative care in elective colorectal surgery: Enhanced Recovery After Surgery (ERAS) Society recommendations 2025. Surgery 184:109397. 10.1016/j.surg.2025.109397 40783294 10.1016/j.surg.2025.109397

[4] Silaghi A, Serban D, Tudor C, Cristea BM, Tribus LC, Shevchenko I et al (2025) A Review of Postoperative Complications in Colon Cancer Surgery: The Need for Patient-Centered Therapy. J Mind Med Sci 12(1):21

[5] Hjortborg M, Edin S, Böckelman C, Kaprio T, Li X, Gkekas I et al (2024) Systemic inflammatory response in colorectal cancer is associated with tumour mismatch repair and impaired survival. Sci Rep 14(1):29738. 10.1038/s41598-024-80803-6 39613865 10.1038/s41598-024-80803-6 PMC11607405

[6] Bona D, Danelli P, Sozzi A, Sanzi M, Cayre L, Lombardo F et al (2023) C-reactive Protein and Procalcitonin Levels to Predict Anastomotic Leak After Colorectal Surgery: Systematic Review and Meta-analysis. J Gastrointest Surg 27(1):166–179. 10.1007/s11605-022-05473-z 36175720 10.1007/s11605-022-05473-z

[7] Negrut RL, Cote A, Feder B, Bodog FD, Maghiar AM (2025) Comparative Prognostic Role of PLR and NLR in Colon Cancer: A Retrospective Analysis of Preoperative Inflammatory Markers. Medicina 61(9):1580 41010971 10.3390/medicina61091580 PMC12471475

[8] Ge X, Cao Y, Wang H, Ding C, Tian H, Zhang X et al (2017) Diagnostic accuracy of the postoperative ratio of C-reactive protein to albumin for complications after colorectal surgery. World J Surg Oncol 15(1):15. 10.1186/s12957-016-1092-1 28069031 10.1186/s12957-016-1092-1 PMC5223565

[9] Halberg N, Schraw TD, Wang ZV, Kim JY, Yi J, Hamilton MP et al (2009) Systemic fate of the adipocyte-derived factor adiponectin. Diabetes 58(9):1961–1970. 10.2337/db08-1750 19581422 10.2337/db08-1750 PMC2731534

[10] Burgos-Molina AM, Tellez Santana T, Redondo M, Bravo Romero MJ (2024) The Crucial Role of Inflammation and the Immune System in Colorectal Cancer Carcinogenesis: A Comprehensive Perspective. Int J Mol Sci 25(11). 10.3390/ijms25116188 10.3390/ijms25116188 PMC11172443 38892375

[11] Vahed IE, Moshgelgosha M, Kor A, Minadi M, Ebrahimi F, Azhdarian A et al (2025) The role of Adiponectin and Leptin in Colorectal Cancer and Adenoma: a systematic review and meta-analysis. BMC Cancer 25(1):968. 10.1186/s12885-025-14362-y 40448255 10.1186/s12885-025-14362-y PMC12123994

[12] Gianoli S, Tang J, Odegard KC, Yuki K, Koutsogiannaki S (2025) Harnessing adiponectin for sepsis: current knowledge, clinical insights and future therapies. Crit Care 29(1):300. 10.1186/s13054-025-05516-2 40652274 10.1186/s13054-025-05516-2 PMC12255986

[13] Tojek K, Anaszewicz M, Szukay B, Czerniak B, Socha E, Lis K et al (2021) Circulating Leptin, Adiponectin, and Tumor Necrosis Factor-Alpha in Patients Undergoing Surgery Due to Colorectal Cancer. Digestion 102(2):246–255. 10.1159/000504507 31747664 10.1159/000504507

[14] Neskovic N, Marczi S, Mandic D, Mraovic B, Skiljic S, Kristek G et al (2021) Analgesic Effect of Tramadol Is Not Altered by Postoperative Systemic Inflammation after Major Abdominal Surgery. Acta Clin Croat 60(2):268–275. 10.20471/acc.2021.60.02.13 34744277 10.20471/acc.2021.60.02.13 PMC8564835

[15] Cicarelli DD, Vieira JE, Bensenor FE (2007) [Lactate as a predictor of mortality and multiple organ failure in patients with the systemic inflammatory response syndrome]. Rev Bras Anestesiol 57(6):630–638. 10.1590/s0034-70942007000600005 19462139 10.1590/s0034-70942007000600005

[16] Castelli GP, Pognani C, Meisner M, Stuani A, Bellomi D, Sgarbi L (2004) Procalcitonin and C-reactive protein during systemic inflammatory response syndrome, sepsis and organ dysfunction. Crit Care 8(4):R234–R242. 10.1186/cc2877 15312223 10.1186/cc2877 PMC522844

[17] Muirhead B, Weiss ADH (2017) Massive hemorrhage and transfusion in the operating room. Can J Anesthesia/Journal canadien d’anesthésie 64(9):962–978. 10.1007/s12630-017-0925-x 10.1007/s12630-017-0925-x 28718098

[18] Polito R, Nigro E, Fei L, L DEM, Monaco ML, D’Amico R et al (2020) Adiponectin Is Inversely Associated With Tumour Grade in Colorectal Cancer Patients. Anticancer Res 40(7):3751–3757. 10.21873/anticanres.14364 32620614 10.21873/anticanres.14364

[19] Parmesh P, Dinesh US, Khandagale AS, Bapu AB, Sadashiv R, Reddy P (2024) Correlation of Leptin and Adiponectin Receptor Expression with Clinicopathological Parameters in Colorectal Carcinoma - a Cross-Sectional Prospective Study. Arq Gastroenterol 61:e24016. 10.1590/S0004-2803.24612024-016 38775586 10.1590/S0004-2803.24612024-016

[20] Parmesh P, Udupi Shastri D, Goni M, Bargale AB, Khandagale AS (2024) mRNA expression profiling of leptin and adiponectin and its receptors in colorectal carcinoma – Biomarker development. Adv Cancer Biology - Metastasis 10:100118. 10.1016/j.adcanc.2024.100118

[21] Faul F, Erdfelder E, Lang AG, Buchner A (2007) G*Power 3: a flexible statistical power analysis program for the social, behavioral, and biomedical sciences. Behav Res Methods 39(2):175–191. 10.3758/bf03193146 17695343 10.3758/bf03193146

[22] El-Hussuna A, Hussain MI, Cuk P, Ellebaek MB (2025) A systematic review on biomarkers implemented to measure surgical stress response in patients undergoing colorectal surgery. Int J Colorectal Dis 40(1):168. 10.1007/s00384-025-04950-6 40760346 10.1007/s00384-025-04950-6 PMC12321657

[23] Kasten-Jolly J, Lawrence DA (2022) Differential blood leukocyte populations based on individual variances and age. Immunol Res 70(1):114–128. 10.1007/s12026-021-09257-6 35023048 10.1007/s12026-021-09257-6 PMC8754550

[24] Edomskis PP, Dik WA, Sparreboom CL, Nagtzaam NMA, van Oudenaren A, Lambrichts DPV et al (2022) Monocyte response after colorectal surgery: A prospective cohort study. Front Immunol 13:1031216. 10.3389/fimmu.2022.1031216 36389839 10.3389/fimmu.2022.1031216 PMC9647000

[25] Wang B, Ling D, Li L, Zhang J, Xu J (2024) Impact of preoperative white blood cell count on outcomes in different stage colorectal cancer patients undergoing surgical resection: a single-institution retrospective cohort study. BMC Cancer 24(1):242. 10.1186/s12885-024-11983-7 38383340 10.1186/s12885-024-11983-7 PMC10882932

[26] Sarbinowski R, Arvidsson S, Tylman M, Oresland T, Bengtsson A (2005) Plasma concentration of procalcitonin and systemic inflammatory response syndrome after colorectal surgery. Acta Anaesthesiol Scand 49(2):191–196. 10.1111/j.1399-6576.2004.00565.x 15715620 10.1111/j.1399-6576.2004.00565.x

[27] Watt DG, Horgan PG, McMillan DC (2015) Routine clinical markers of the magnitude of the systemic inflammatory response after elective operation: a systematic review. Surgery 157(2):362–380. 10.1016/j.surg.2014.09.009 25616950 10.1016/j.surg.2014.09.009

[28] Ma M, Jiang W, Zhou R (2024) DAMPs and DAMP-sensing receptors in inflammation and diseases. Immunity 57(4):752–771. 10.1016/j.immuni.2024.03.002 38599169 10.1016/j.immuni.2024.03.002

[29] Paruk F, Chausse JM (2019) Monitoring the post surgery inflammatory host response. J Emerg Crit Care Med. 3:47. 10.21037/jeccm.2019.08.06

[30] de Bois J, Moor D, Aggarwal G (2023) Systemic response to surgery. Surg (Oxford) 41(2):117–121. 10.1016/j.mpsur.2022.12.001

[31] Bain CR, Myles PS, Corcoran T, Dieleman JM (2023) Postoperative systemic inflammatory dysregulation and corticosteroids: a narrative review. Anaesthesia 78(3):356–370. 10.1111/anae.15896 36308338 10.1111/anae.15896 PMC10092416

[32] Neskovic N, Mandic D, Marczi S, Skiljic S, Kristek G, Vinkovic H et al (2021) Different Pharmacokinetics of Tramadol, O-Demethyltramadol and N-Demethyltramadol in Postoperative Surgical Patients From Those Observed in Medical Patients. Front Pharmacol 12:656748. 10.3389/fphar.2021.656748 33935773 10.3389/fphar.2021.656748 PMC8082457

[33] Choi HM, Doss HM, Kim KS (2020) Multifaceted Physiological Roles of Adiponectin in Inflammation and Diseases. Int J Mol Sci 21(4). 10.3390/ijms21041219 10.3390/ijms21041219 PMC7072842 32059381

[34] Salvator H, Grassin-Delyle S, Brollo M, Couderc LJ, Abrial C, Victoni T et al (2021) Adiponectin Inhibits the Production of TNF-α, IL-6 and Chemokines by Human Lung Macrophages. Front Pharmacol 12:718929. 10.3389/fphar.2021.718929 34512346 10.3389/fphar.2021.718929 PMC8428996

[35] Asgeirsson T, Zhang S, Khoo SK, Resau JH, Dujovny N, Senagore AJ (2011) Serum adiponectin, resistin, and circulating soluble receptor for advanced glycation end products in colectomy patients. Mediators Inflamm 2011:916807. 10.1155/2011/916807 21912448 10.1155/2011/916807 PMC3168899

[36] Ekstrom M, Soderberg S, Tornvall P (2015) Acute Systemic Inflammation is Unlikely to Affect Adiponectin and Leptin Synthesis in Humans. Front Cardiovasc Med 2:7. 10.3389/fcvm.2015.00007 26664879 10.3389/fcvm.2015.00007 PMC4671352

[37] Shi W, Lou J, Zhang X, Ji Y, Weng X, Du J (2021) Adipose tissue alleviates the stress response by releasing adiponectin during laparoscopic surgery in patients with colorectal cancer. Lipids Health Dis 20(1):166. 10.1186/s12944-021-01595-6 34801038 10.1186/s12944-021-01595-6 PMC8606056

[38] Yamamoto H, Maeda K, Arima H, Sonoda H, Shimizu T, Mekata E et al (2016) Perioperative Adiponectin Measurement is Useful for Prediction of Postoperative Infection in Patients with Colorectal Cancer. Ann Surg Oncol 23(4):540–545. 10.1245/s10434-016-5386-x 27364509 10.1245/s10434-016-5386-x

[39] Ortega-Deballon P, Menegaut L, Fournel I, Orry D, Masson D, Binquet C et al (2015) Are Adiponectin and Leptin Good Predictors of Surgical Infection after Colorectal Surgery? A Prospective Study. Surg Infect (Larchmt) 16(5):566–571. 10.1089/sur.2014.206 26114869 10.1089/sur.2014.206

[40] Matsuda A, Matsutani T, Sasajima K, Furukawa K, Tajiri T, Tamura K et al (2009) Preoperative plasma adiponectin level is a risk factor for postoperative infection following colorectal cancer surgery. J Surg Res 157(2):227–234. 10.1016/j.jss.2008.09.007 19394964 10.1016/j.jss.2008.09.007

[41] Widmar M, Keskin M, Strombom PD, Gennarelli RL, Szeglin BC, Smith JJ et al (2021) Evaluating the Validity of the Clavien-Dindo Classification in Colectomy Studies: A 90-Day Cost of Care Analysis. Dis Colon Rectum 64(11):1426–1434. 10.1097/DCR.0000000000001966 34623350 10.1097/DCR.0000000000001966 PMC8502230

[42] Smeyers K, Slankamenac K, Houben B, Sergeant G (2022) Comparison of the Clavien-Dindo and Comprehensive Complication Index systems for grading of surgical complications after colorectal resections. Acta Chir Belg 122(6):403–410. 10.1080/00015458.2021.1920682 33910493 10.1080/00015458.2021.1920682

[43] Kim YS, Yang SY, Kim NR, Kim IK, Jung EJ, Kim YM et al (2026) Comparing the Comprehensive Complication Index and Clavien-Dindo classification for evaluating postoperative complication severity in major abdominal surgery. Surgery 190:109924. 10.1016/j.surg.2025.109924 41349191 10.1016/j.surg.2025.109924

[44] Gwenzi T, Schrotz-King P, Anker SC, Schottker B, Hoffmeister M, Brenner H (2024) Post-operative C-reactive protein as a strong independent predictor of long-term colorectal cancer outcomes: consistent findings from two large patient cohorts. ESMO Open 9(4):102982. 10.1016/j.esmoop.2024.102982 38613909 10.1016/j.esmoop.2024.102982 PMC11033061

[45] Chelazzi C, Villa G, Mancinelli P, Becatti M, Boddi M, Coppo M et al (2017) Perioperative Levels of Adiponectin and Oxidative Stress in Patients Undergoing Laparoscopic Abdominal Surgery for Cancer. Antiinflamm Antiallergy Agents Med Chem 16(3):168–174. 10.2174/1871523016666171114164219 29141566 10.2174/1871523016666171114164219

[46] McSorley ST, Watt DG, Horgan PG, McMillan DC (2016) Postoperative Systemic Inflammatory Response, Complication Severity, and Survival Following Surgery for Colorectal Cancer. Ann Surg Oncol 23(9):2832–2840. 10.1245/s10434-016-5204-5 27016295 10.1245/s10434-016-5204-5 PMC4972846

[47] Kim M, Kim H, Kim K, Cho J, Jeong W, Baek S et al (2025) Effect of Preoperative Inflammatory Diet on Clinical and Oncologic Outcomes Following Colorectal Cancer Surgery. Nutrients 17(9). 10.3390/nu17091522 10.3390/nu17091522 PMC12074250 40362831

[48] Kampman SL, Smalbroek BP, Dijksman LM, Smits AB (2023) Postoperative inflammatory response in colorectal cancer surgery: a meta-analysis. Int J Colorectal Dis 38(1):233. 10.1007/s00384-023-04525-3 37725227 10.1007/s00384-023-04525-3

[49] Prochazka V, Lacina L, Smetana K Jr., Svoboda M, Skrivanova K, Benovska M et al (2023) Serum concentrations of proinflammatory biomarker interleukin-6 (IL-6) as a predictor of postoperative complications after elective colorectal surgery. World J Surg Oncol 21(1):384. 10.1186/s12957-023-03270-9 38098074 10.1186/s12957-023-03270-9 PMC10720211

[50] Rettig TC, Verwijmeren L, Dijkstra IM, van de Boerma D, Noordzij PG (2016) Postoperative Interleukin-6 Level and Early Detection of Complications After Elective Major Abdominal Surgery. Ann Surg 263(6):1207–1212. 10.1097/SLA.0000000000001342 26135695 10.1097/SLA.0000000000001342

[51] Boersema GSA, Wu Z, Menon AG, Kleinrensink GJ, Jeekel J, Lange JF (2018) Systemic Inflammatory Cytokines Predict the Infectious Complications but Not Prolonged Postoperative Ileus after Colorectal Surgery. Mediators Inflamm 2018:7141342. 10.1155/2018/7141342 29692682 10.1155/2018/7141342 PMC5859856

[52] Martin D, Hubner M, Moulin E, Pache B, Clerc D, Hahnloser D et al (2018) Timing, diagnosis, and treatment of surgical site infections after colonic surgery: prospective surveillance of 1263 patients. J Hosp Infect 100(4):393–399. 10.1016/j.jhin.2018.09.011 30266537 10.1016/j.jhin.2018.09.011

[53] Su’a B, Tutone S, MacFater W, Barazanchi A, Xia W, Zeng I et al (2020) Diagnostic accuracy of procalcitonin for the early diagnosis of anastomotic leakage after colorectal surgery: a meta-analysis. ANZ J Surg 90(5):675–680. 10.1111/ans.15291 31230412 10.1111/ans.15291

[54] Jung HN, Jung CH (2021) The Role of Anti-Inflammatory Adipokines in Cardiometabolic Disorders: Moving beyond Adiponectin. Int J Mol Sci 22(24). 10.3390/ijms222413529 10.3390/ijms222413529 PMC8707770 34948320

[55] Milone M, Desiderio A, Velotti N, Manigrasso M, Vertaldi S, Bracale U et al (2021) Surgical stress and metabolic response after totally laparoscopic right colectomy. Sci Rep 11(1):9652. 10.1038/s41598-021-89183-7 33958669 10.1038/s41598-021-89183-7 PMC8102592

[56] Acharya K, Rout DK, Kapadia NA, Caroicar YS, Karunakaran NB, Patel P et al (2025) Surgical Stress Response: A Physiological Review of the Endocrine, Immune, and Metabolic Changes. Cureus 17(12):e100101. 10.7759/cureus.100101 41589142 10.7759/cureus.100101 PMC12831963

[57] Ramesh SK, Swain SK, Munikrishnan V, Jameel JKA (2023) Can the Inflammatory Cell Ratio NLR and PLR be Used as a Reliable Marker in Colon Cancer? A Prospective Study. Euroasian J Hepatogastroenterol 13(2):61–65. 10.5005/jp-journals-10018-1399 38222963 10.5005/jp-journals-10018-1399 PMC10785127

[58] Fagarasan G, Seicean R, Bintintan V, Fagarasan V, Caziuc A, Andras D et al (2024) The Value of Preoperative C-Reactive Protein to Albumin Ratio as a Prognostic Biomarker in Colon Cancer Patients. Medicina 60(7):1054 39064483 10.3390/medicina60071054 PMC11278571

[59] Xu N, Zhang JX, Zhang JJ, Huang Z, Mao LC, Zhang ZY et al (2025) The prognostic value of the neutrophil-to-lymphocyte ratio (NLR) and platelet-to-lymphocyte ratio (PLR) in colorectal cancer and colorectal anastomotic leakage patients: a retrospective study. BMC Surg 25(1):57. 10.1186/s12893-024-02708-5 39910526 10.1186/s12893-024-02708-5 PMC11796187

[60] Paliogiannis P, Deidda S, Maslyankov S, Paycheva T, Farag A, Mashhour A et al (2020) Blood cell count indexes as predictors of anastomotic leakage in elective colorectal surgery: a multicenter study on 1432 patients. World J Surg Oncol 18(1):89. 10.1186/s12957-020-01856-1 32375770 10.1186/s12957-020-01856-1 PMC7204308

[61] Donlon NE, Mohan H, Free R, Elbaghir B, Soric I, Fleming C et al (2020) Predictive value of CRP/albumin ratio in major abdominal surgery. Ir J Med Sci (1971 -) 189(4):1465–1470. 10.1007/s11845-020-02238-y 10.1007/s11845-020-02238-y 32361882

[62] Bohne A, Grundler E, Knuttel H, Volkel V, Furst A (2024) Impact of laparoscopic versus open surgery on humoral immunity in patients with colorectal cancer: a systematic review and meta-analysis. Surg Endosc 38(2):540–553. 10.1007/s00464-023-10582-0 38102395 10.1007/s00464-023-10582-0 PMC10830603

